# Pure somatic pathogenic variation profiles for patients with serrated polyposis syndrome: a case series

**DOI:** 10.1186/s13104-022-06245-3

**Published:** 2022-11-22

**Authors:** Misaki Hidaka, Moriya Iwaizumi, Terumi Taniguchi, Satoshi Baba, Satoshi Osawa, Ken Sugimoto, Masato Maekawa

**Affiliations:** 1 Department of Laboratory Medicine, Hamamatsu University School of Medicine, 1-20-1 Handayama, Higashi-Ku, Hamamatsu, 431-3192 Japan; 2 Department of Diagnostic Pathology, Hamamatsu University Hospital, Hamamatsu, Japan; 3 Department of Endoscopic and Photodynamic Medicine, Hamamatsu University of School of Medicine, Hamamatsu, Japan; 4 First Department of Medicine, Hamamatsu University School of Medicine, Hamamatsu, Japan

**Keywords:** Serrated polyposis syndrome, Somatic pathogenic variant, Serrated pathway

## Abstract

**Objective:**

The serrated pathway is a distinct genetic/epigenetic mechanism of the adenoma-carcinoma sequence in colorectal carcinogenesis. Although many groups have reported the genetic-phenotypic correlation of serrated lesions (SLs), previous studies regarding the serrated pathway were conducted on patients with SLs that have different germline and environmental genetic backgrounds. We aimed to compare pure somatic genetic profiles among SLs within identical patient with SPS.

**Results:**

We analyzed SLs from one patient with SPS (Case #1) and compared DNA variant profiles using targeted DNA multigene panels via NGS among the patient’s hyperplastic polyp (HP), three sessile serrated lesions (SSLs), and one traditional serrated adenoma (TSA), and separately analyzed three SSLs and one tubular adenoma (TA) within another patient with SPS (Case #2). In two patients, known pathogenic variant of *BRAF* (c.1799 T > A, p.Val600Glu) was observed in one TSA and one SSL in Case #1, and in three SSLs within Case #2. The pure somatic pathogenic variant *BRAF* (c.1799 T > A, p.Val600Glu) among SLs with identical germline genetic background supports its importance as a strong contributor for SLs.

**Supplementary Information:**

The online version contains supplementary material available at 10.1186/s13104-022-06245-3.

## Introduction

Colorectal cancer (CRC) is one of the most common cancers worldwide and ranks as the sixth leading cause of cancer-related deaths [1, 2]. Since CRC arises from premalignant polyps, the detection and removal of these lesions decreases both CRC incidence and mortality [3]. Some groups have reported that 15–30% of all CRCs are initiated from serrated lesions (SLs) rather than conventional adenomas arising through the adenoma-carcinoma sequence [4–6]. SLs are histologically heterogeneous, including benign hyperplastic polyps (HPs), precancerous sessile serrated lesions (SSLs), or traditional serrated adenomas (TSAs) [4]. Among these SLs, HPs are the most frequent subtype and SSLs are the second most common form of SLs. SSLs are recognized as important precursors of the serrated pathway showing a high CpG island methylator phenotype (CIMP) [7, 8].

The serrated pathway is a distinct genetic/epigenetic mechanism of colorectal carcinogenesis, but this has not been fully characterized. Although many groups have reported the genetic-phenotypic correlation regarding SLs, the precise profile and mechanisms of these serrated pathways for the prevention of colorectal carcinogenesis are not fully elucidated, as previous reports involved many patients with SLs with different germline and environmental backgrounds [9–21]. Therefore, genetic comparison and analysis of multiple SLs within the same patient should be conducted.

Serrated polyposis syndrome (SPS) is characterized by multiple SLs located throughout the colon and is accompanied by an increased risk of CRC. The diagnosis of SPS is based on the cumulative lifetime number of HPs, TSAs, and SSPs in a patient who meets one of the two following World Health Organization (WHO) criteria, including (1) > 5SPs proximal to the rectum, all being ≥ 5 mm in size, including ≥ 2 that are ≥ 10 mm; or (2) > 20 SPs of any size distributed throughout the colon, with ≥ 5 being proximal to the rectum [22]. Therefore, it is important to compare genetic profiles among SLs within identical patient with SPS to understand the pure somatic genetic variant associated with the serrated pathway.

In the present study, we customized a set of targeted DNA multigene panels and used it to evaluate the variant SL profiles. Herein, we show differences in the main genetic contributors to the serrated pathway among SLs within the same SPS patient.

## Main text

### Methods

#### Patients

We analyzed nine SLs and one non-SL (tubular adenoma) from two patients with SPS who met the WHO 2019 criteria for the diagnosis of SPS. Both patients provided written informed consent, and the study was approved by the Institutional Review Board of the Hamamatsu University School of Medicine (Approval No. 17–222).

#### Samples, DNA extraction, and quality assessment

SL samples were obtained from the Department of Diagnostic Pathology at Hamamatsu University Hospital as formalin-fixed paraffin-embedded (FFPE) tissue from the two patients with SPS. The polyps were resected by EMR. We also obtained matched normal blood samples. Genomic DNA was extracted from macrodissected tumorous and non-tumorous tissue using a QIAamp DNA FFPE Tissue Kit (Qiagen, Hilden, Germany), and extracted from the blood using an EZ1 DNA Blood 350 µl Kit (Qiagen). The quality of the gDNA was analyzed using the 2200 TapeStation (Agilent Technologies, Santa Clara, CA, USA) system using the TapeStation Analysis software (Agilent), which automatically determines and displays the DNA integrity number (DIN) as a measure of DNA integrity (https://www.agilent.com/cs/library/applications/5991-5258EN.pdf).

#### Next-generation sequencing (NGS)

We customized the multigene panel (72 genes) by adding the QIAseq Human Colorectal Cancer Panel (71 genes, DHS-002Z; Qiagen) to the *RNF43* gene because the pathogenicity of the *RNF43* gene variant has been reported in SLs [23]. The customized multigene panel was used for library construction according to the manufacturer’s instructions. The libraries were assessed using a QIAseq Library Quant Assay Kit (#QSTF-ILZ-R; Qiagen) and applied to a MiniSeq sequencer (Illumina, San Diego, CA, USA). The Qiagen web portal (https://geneglobe.qiagen.com/jp/analyze/) and VariantStudio software (Illumina) were used for data analysis and alignment. GRCH37 was used as the reference genome. All detected variants were validated using Integrative Genomics Viewer 2.9.2 (IGV; http://software.broadinstitute.org/software/igv/home).

#### IHC staining

IHC was performed as described previously [24].

#### Statistical analyses

Statistical analyses were performed using IBM SPSS Statistics for Windows (version 25; IBM Corp., Armonk, NY, USA), and a value of *P* < 0.05 was considered statistically significant.

## Results

### Clinicopathological features

**Fig. 1:**
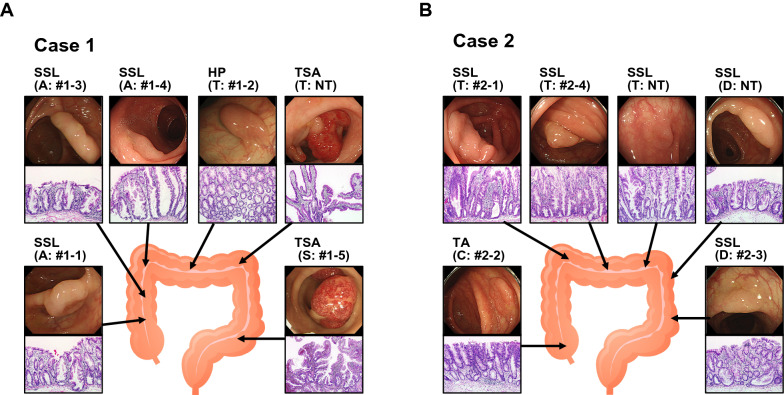
Endoscopic/histological features of analyzed lesions. A Case #1. A man who initially exhibited a positive fecal occult blood test (FOBT). He underwent colonoscopy and six protruded lesions were detected throughout the colon, and SLs (one HP, three SSLs, and two TSAs) were diagnosed histopathologically in each resected specimen. **B** Case #2. A woman who underwent colonoscopy as part of a routine medical examination, and six protruded lesions were detected throughout the colon. Among the lesions, five were histologically SSLs, and one was non-SL (tubular adenoma). SSL sessile serrated lesion, HP hyperplastic polyp, TSA traditional serrated adenoma, TA tubular adenoma, A Ascending Colon, T Transverse Colon, S Sigmoid Colon, C Cecum, D Descending Colon, NT not tested

We analyzed SLs from two patients with SPS (Cases #1 and #2). A man (Case #1) underwent colonoscopy and six protruded lesions were detected throughout the colon (Fig. 1). We performed endoscopic mucosal resection (EMR) on all lesions, and SLs (one HP in the transverse colon, three SSLs in the ascending colon, and two TSAs in the transverse colon and the sigmoid colon, respectively) were diagnosed histopathologically in each resected specimen. Case #2 is a woman who underwent colonoscopy as part of a routine medical examination, and six protruded lesions were detected throughout the colon. All lesions were located proximal to the rectum (Fig. 1) and were endoscopically resected. Among the lesions, five were histologically SSLs (three lesions in the transverse colon and two in the descending colon), and one was non-SL (tubular adenoma) in the cecum. Both two patients were diagnosed with SPS because they met the WHO 2019 criteria.

### Somatic variant profile of analyzed lesions from patients with SPS

The patient in Case #1 exhibited all pathological SL types (HP, SSL, and TSA) and we analyzed the DNA variant profile of one HP (three SSLs, and one TSA (Table 1, Additional file 1: Table S1). When focusing on gene variants known to be associated with SLs, a known pathogenic variant of *BRAF* (c.1799 T > A, p.Val600Glu) was detected in one SSL located in the ascending colon (#1–4) and one TSA in the transverse colon (#1–5) among the six SLs. One SSL in the ascending colon displayed a splice site variant at *RNF43 *(c.687G > A) without any *BRAF* variant. The SSL with the *BRAF* c.1799 T > A pathogenic variant located in the ascending colon also displayed the *MLH1* variant (c.687G > A, p.Val213Glu).

**Table 1:** DNA variant profile (Case #1)

Gene	DNA variant	Amino acid alteration	Variant allele frequency (%) **(Bold: VAF > 5%)**					
			#1–0 blood	#1–1 SSL(A)	#1–2 HP(T)	#1–3 SSL(A)	#1–4 SSL(A)	#1–5 TSA(S)
*DCC*	NM_005215.3:c.2277 T > G	NP_005206.2:p.Ile759Met	**47.3**	**53.5**	**54.2**	**50.8**	**50.9**	**54.9**
*MSH3*	NM_002439.4:c.1718G > A	NP_002430.3:p.Arg573Lys	**51.1**	**52.9**	**81.8**	**42.3**	**58.6**	**50**
*BAX*	NM_004324.3:c.32G > A	NP_004315.1:p.Gly11Glu	**35.9**	**46.7**	**42.9**	**45.7**	**49.5**	**49.0**
*SRC*	NM_005417.4:c.532C > T	NP_005408.1:p.Arg178Ter	0	**6.0**	0	0	0	0
*RET*	NM_020975.4:c.296G > A	NP_066124.1:p.Arg99Gln	0	**6.7**	0	0	0	0
*MLH3*	NM_001040108.1:c.3769 T > C	NP_001035197.1:p.Ser1257Pro	0	**11.5**	0	0	0	0
*TCERG1*	NM_006706.3:c.1705G > C	NP_006697.2:p.Asp569His	0	**25.7**	0	0	0	0
*BUB1B*	NM_001211.5:c.898A > C	NP_001202.4:p.Met300Leu	0	0	**18.8**	0	0	0
*PALB2*	NM_024675.3:c.829G > A	NP_078951.2:p.Asp277Asn	0	0	**30.0**	0	0	0
*CHEK2*	NM_001005735.1:c.1696C > T	NP_001005735.1:p.Arg566Cys	0	0	**16.7**	0	0	0
*CTNNA1*	NM_001903.2:c.2281C > T	NP_001894.2:p.Arg761Cys	0	0	**15.8**	0	0	0
*TCF7L2*	NM_001146274.1:c.1001C > T	NP_001139746.1:p.Ser334Leu	0	0	**6.2**	0	0	0
*ATM*	NM_000051.3:c.5189G > A	NP_000042.3:p.Arg1730Gln	0	0	0	**7.9**	0	0
*TP53*	NM_000546.5:c.818G > T	NP_000537.3:p.Arg273Leu	0	0	0	**9.1**	0	0
*RNF43*	NM_017763.4:c.687G > A	splicing site	0	0	0	**7.4**	0	0
*BLM*	NM_000057.2:c.1544delA	NP_000048.1:p.Asn515MetfsTer16	0	0	0	0	**9.1**	0
*AXIN2*	NM_004655.3:c.1419_1421delCCA	NP_004646.3:p.His474del	0	0	0	0	**6.1**	0
*CDC27*	NM_001114091.1:c.1750A > G	NP_001107563.1:p.Ser584Gly	0	0	0	0	**5.8**	0
*CDC27*	NM_001114091.1:c.1665 T > G	NP_001107563.1:p.Asp555Glu	0	0	0	0	**7.4**	0
*MLH1*	NM_000249.3:c.638 T > A	NP_000240.1:p.Val213Glu	0	0	0	0	**8.3**	0
*BRAF*	NM_004333.4:c.1799 T > A	NP_004324.2:p.Val600Glu	0	0	0	0	**14.7**	**13.9**
*RET*	NM_020975.4:c.2842G > A	NP_066124.1:p.Gly948Arg	0	0	0	0	0	**6.3**
*ERBB2*	NM_004448.2:c.1295G > A	NP_004439.2:p.Arg432Gln	0	0	0	0	0	**10.5**
*ERBB2*	NM_004448.2:c.1846 T > C	NP_004439.2:p.Phe616Leu	0	0	0	0	0	**5.7**
*STK11*	NM_000455.4:c.928C > T	NP_000446.1:p.Arg310Trp	0	0	0	0	0	**6.7**
*TCERG1*	NM_006706.3:c.2870delA	NP_006697.2:p.Lys957ArgfsTer17	0	0	0	0	3.5	**11.3**

*HP* hyperplastic polyp, *SSL* sessile serrated lesion, *TSA* traditional serrated adenoma, *A* Ascending Colon, *T* Transverse Colon, *S* Sigmoid Colon

The patient in Case #2 had only one type of SL (three SSLs), but it is unique that we could compare the DNA profile of three SSLs with that of one non-SL lesion (tubular adenoma) (Table 2, Additional file 2: Table S2). A known pathogenic variant of *BRAF* (c.1799 T > A, p.Val600Glu) was detected in all SSLs analyzed (#2–1, #2–3, and #2–4), whereas we detected another two *BRAF* variants, not known to be pathogenic in the previous database, in tubular adenomas of patients. No *KRAS* or *RNF43* variants were detected among the four lesions, including tubular adenomas. Interestingly, a tubular adenoma displayed two pathogenic variants that are highly associated with the adenoma-carcinoma sequence (*APC*; c.4249_4265delATTATAAGCCCCAGTGA, p.Ile1417SerfsTer4, *TP53*; c.818G > A, p.Arg273His), but not the other three SSLs. Among all the nine lesions, we detected no lesions with defective MLH1 proteins by IHC.

**Table 2:** DNA variant profile (Case #2)

Gene	DNA variant	Amino acid alteration	Variant allele frequency (%) **(Bold: VAF > 5%)**				
			#2–0 Blood	#2–1 SSL(T)	#2–2 TA(C)	#2–3 SSL(D)	#2–4 SSL(T)
*BRCA2*	NM_000059.3:c.2350A > G	NP_000050.2:p.Met784Val	**49.3**	**56.5**	**46.4**	**45.5**	**51.5**
*BRCA2*	NM_000059.3:c.3420 T > A	NP_000050.2:p.Ser1140Arg	**53.7**	**52.9**	**50.0**	**51.9**	**50.3**
*BRCA1*	NM_007300.3:c.670 + 1G > T	splicing site	**58.9**	**57.1**	**50.0**	**55.0**	**53.5**
*AXIN2*	NM_004655.3:c.2140C > T	NP_004646.3:p.Arg714Trp	**43.8**	**44.4**	**36.7**	**44.3**	**49.8**
*MET*	NM_001127500.1:c.4141G > A	NP_001120972.1:p.Ala1381Thr	**48.6**	**48.4**	**49.4**	**46.6**	**55.8**
*BRAF*	NM_004333.4:c.1799 T > A	NP_004324.2:p.Val600Glu	0	**15.8**	0	**10.6**	**9.5**
*ERBB2*	NM_004448.2:c.1846 T > C	NP_004439.2:p.Phe616Leu	0	**6.6**	0	0	0
*DMD*	NM_004006.2:c.8851C > A	NP_003997.1:p.Arg2951Ser	0	**6.9**	0	0	0
*APC*	NM_000038.5: c.4249_4265delATTATAAGCCCCAGTGA	NP_000029.2:p.Ile1417SerfsTer4	0	0	**55.4**	0	0
*TP53*	NM_000546.5:c.818G > A	NP_000537.3:p.Arg273His	0	0	**27.9**	0	0
*MSH2*	NM_000251.2:c.727C > T	NP_000242.1:p.Arg243Trp	0	0	**10.5**	0	0
*CTNNB1*	NM_001904.3:c.1267A > T	NP_001895.1:p.Ile423Phe	0	0	**34.6**	0	0
*FBXW7*	NM_033632.3:c.227A > T	NP_361014.1:p.Gln76Leu	0	0	**24.5**	0	0
*BRAF*	NM_004333.4:c.1781A > G	NP_004324.2:p.Asp594Gly	0	0	**38.5**	0	0
*BRAF*	NM_004333.4:c.1085G > A	NP_004324.2:p.Arg362Gln	0	0	**26.4**	0	0
*GALNT12*	NM_024642.4:c.1250G > A	NP_078918.3:p.Arg417Gln	0	0	**22.4**	0	0
*FGFR3*	NM_001163213.1:c.929A > C	NP_001156685.1:p.Lys310Thr	0	0	**10.3**	0	0
*FGFR3*	NM_001163213.1:c.930 + 5G > C	splicing site	0	0	**11.5**	0	0
*CDC27*	NM_001114091.1:c.1750A > G	NP_001107563.1:p.Ser584Gly	0	0	0	0	**6.2**
*CDC27*	NM_001114091.1:c.1060C > A	NP_001107563.1:p.Gln354Lys	0	0	0	0	**5.5**
*CDC27*	NM_001114091.1:c.1039G > A	NP_001107563.1:p.Glu347Lys	0	0	0	0	**5.5**
*CDC27*	NM_001114091.1:c.80 T > C	NP_001107563.1:p.Leu27Pro	0	0	0	0	**5.2**
*CDC27*	NM_001114091.1:c.77 T > C	NP_001107563.1:p.Phe26Ser	0	0	0	0	**6.5**

*SSL* sessile serrated lesion, *TA* tubular adenoma, *C* Cecum, *A* Ascending Colon, *T* Transverse Colon, *D* Descending Colon

### Mutational signature patterns

Mutational signatures (MS) were analyzed by examining combinations of single base substitutions and further including flanking 5’ and 3’ bases of each mutated site. As shown in Additional file 3: Table S3, the most common type of single base substitution (SBS) was C > T, followed by T > C among nine SSLs. Especially, C > T SBS tended to be commonly observed in HP (#1–2) and TSA (#1–5) of case 1 and TA (#2–2) of case 2, and T > C occurred in one SSL (#2–4) of case 2. It is possible that both non-SSL serrated legions such as HP, TSA, and non-SLs as TA, may be characterized as MS patterns seen by aging. Additionally, among nine regions, we observed nine substitutional sets of CCG > CTG, six sets of TCG > TTG and GTG > GAG, and five sets of ACC > AGC and GCT > GGT, but a typical MS pattern was not identified (Additional file 4: Table S4).

## Discussion

Some groups have reported on the molecular characteristics of various types of serrated lesions. However, the collected tumor samples had various genetic germline backgrounds and were obtained from patients who were subjected to different environmental factors, lifestyles and microbiomes. Therefore, molecular analysis should be performed using SLs from patients with identical genetic backgrounds. Our study demonstrated that (a) favorable DNA samples (≥ 4.0 DIN) can be obtained from FFPE tissues stored for 2 years or more to detect appropriate somatic DNA profiles using NGS, (b) pure somatic SL DNA profiles within a SPS patient were compatible with previous SL reports using patients with heterogeneous germline genetic backgrounds, and (c) pure DNA profiles of TA are quite different from that of other SLs within a patient with SPS. To our knowledge, this is the first study to demonstrate a pure somatic genetic profile compared among SLs within the same patient.

Many groups have reported the influence of pathological genetic variations of *BRAF*, such as c.1799 T > A and p.Val600Glu, on the progression of HPs, SSLs, TSAs, and *KRAS* pathogenic variants for HPs and TSAs, but these analyses were performed among patients with heterogeneous germline backgrounds [10–13]. To detect a pure somatic genetic variation profile, we compared the genetic profiles of dome-serrated lesions within the same identical patient. In Case #1, a known pathogenic variant of *BRAF* (c.1799 T > A, p.Val600Glu) was detected in one SSL (#1–4) and one TSA (#1–5). Previous reports have demonstrated that the *BRAF* variant was found in almost all SSLs. Accordingly, we detected the *BRAF* variant in two different SLs in patient #1 [12, 13, 20, 21]. When focusing on the two lesions, it is interesting that genetic profiles, other than that of the *BRAF* variant, appear quite different (*BLM*, *AXIN2*, *CDC27*, and *MLH1* in #1–4, and *RET*, *ERBB2*, *STK11*, and *TCERG1* in #1–5, as seen in Additional file 4: Table S4). Therefore, the two SLs must be initiated by the common *BRAF* pathogenic variant, followed by progression via the accumulation of different genetic profiles, but further accumulated findings should be considered. In Case #2, all SSLs displayed pathogenic variants of *BRAF* (c.1799 T > A, p.Val600Glu), as expected from previous reports [14]. In addition, it is interesting that we detected a somatic *APC* deletion (c.4249_4265delATTATAAGCCCCAGTGA) as a driver variant (VAF: 55.4%) in TA (#2–2). Notably, the somatic genetic profile of the TA was quite different from other SSLs within Case #2 (#2–1, #2–3, #2–4), which indicates that the serrated pathway and adenoma-carcinoma sequence do not have common driver variants at the initiation stage, and that the accumulated genetic variant profile is distinct between the two pathways.

*RNF43* has been reported as one of the key genes when pathogenic germline or somatic variants are detected in SLs [23, 25, 26]. Giannakis et al. demonstrated that somatic mutations in *RNF43* occur in 18.9–17.6% of CRC cases, and the majority of *RNF43* somatic mutations were truncating events. Taken together, it is possible that the somatic *RNF43* splice-site variant detected in our study in SSLs of Case #1 (#1–3) is pathogenic in the serrated polyposis-cancer sequence, although additional questions remain as limitations, such as the existence of two hits for the lesion by genetic or epigenetic alteration.

As for epigenetic features in SLs, it has been reported that silencing of *MLH1* plays an important role in the progression of SLs, especially with the *BRAF* pathogenic variant [4, 16], but in our IHC study, no deficiency of MLH1 protein could be seen among SLs in two patients with SPS. Apparently, this result does not agree with a previous report, but it is not clear whether *MLH1* was silenced to completely suppress the expression of MLH1 protein. Moreover, it must be noted that previous clinical reports have demonstrated that deficient-MMR has not been identified in HPs, TSAs, or SSLs, but has been reported in SSL with dysplasia (SSLD) only [21, 27]. Additionally, SSLD is the only pre-cancerous colorectal lesion in which *MLH1* is methylated [28]. Although low sensitivity of the IHC cannot be excluded, it is possible that MLH1 may have not been methylated yet in SSLs without dysplasia. Regarding the occurrence of deficient MMR, patients with pre-cancerous lesions, especially with SSLD, require careful surveillance after resection.

In conclusion, the identification of a pure somatic pathogenic variant of *BRAF* (c.1799 T > A, p.Val600Glu), which was observed among SLs with an identical germline genetic and environmental background, highlights the importance of this variant as a strong contributor for SLs.

## Limitations

The present study has several limitations: i) IHC for DNA MMR was performed only for pre-cancerous lesions and not cancerous lesions, because cancer was not found in the two analyzed SPS patients; ii) the analyzed number of patients with SPS was small; and iii) the methylation profile was not evaluated. These findings require further investigation in future studies.

## Supplementary Information

**Additional file 1: Table S1.** DNA variant profile (Case #1).

**Additional file 2: Table S2.** DNA variant profile (Case #2).

**Additional file 3: Table S3.** Mutational signature of the single base substitutions (SBS).

**Additional file 4: Table S4.** Mutational signature of the single base substitution.

## Data Availability

All data relevant to the study are included in the article.

## Abbreviations

CRC: Colorectal cancer
SL: Serrated lesion
HP: Hyperplastic polyp
SSL: Sessile serrated lesion
TSA: Traditional serrated adenoma
MAP: Mitogen-activated protein
CIMP: CpG island methylator phenotype
MSI: Microsatellite instability
MSI-H: MSI-high
SPS: Serrated polyposis syndrome
WHO: World Health Organization
EMR: Endoscopic mucosal resection
MMR: DNA mismatch repair
SSLD: SSL with dysplasia
